# “Together on the Way”: Occupational Therapy in Mainstream Education—A Narrative Study of Emerging Practice in Switzerland

**DOI:** 10.1155/2019/7464607

**Published:** 2019-04-30

**Authors:** Angelika Echsel, Lee Price, Staffan Josephsson, Christina Schulze

**Affiliations:** ^1^Tschoemp Ergotherapie für Kinder, Naefels, Switzerland; ^2^Zurich University of Applied Sciences, Winterthur, Switzerland; ^3^University of Brighton, Eastbourne, UK; ^4^Karolinska Institutet, Stockholm, Sweden; ^5^Norwegian University of Science and Technology, Trondheim, Norway

## Abstract

In Switzerland, recent changes in legislation have reformed special needs education; more children with special needs are now integrated into mainstream schools. Health professionals such as occupational therapists are not embedded in the Swiss education system, but pediatric occupational therapists are starting to work at schools, with the aim of enabling children's full participation as school students. This is bringing a change to the practice of pediatric occupational therapists. Cultural, political, and social factors differ in many ways from those of other countries where most of the current research on pediatric occupational therapists in mainstream education has been conducted. The need for school-based research that is situated within the political, structural, and cultural context of a country has been stressed in different studies. This qualitative study employed narrative analysis to explore the practice experiences and clinical reasoning of Swiss pediatric occupational therapists when working with children with special needs in the school context. Three main themes were identified in the narratives: “bringing in an occupational therapy perspective,” “focusing on school-related occupations,” and “collaborating with different inclusion players.” These represent three different aspects of the therapists' emerging practice. The participants highlight different approaches for children with special needs to enable their participation in everyday life at school through learning, playing, and being with their peers. The findings are discussed in relation to current international research and with respect to European countries with a similar political and structural context, thus complementing approaches to school-based occupational therapy.

## 1. Introduction

School is a major area of participation and productivity for young people. It is the place where they learn, play, do arts and crafts, engage in sports, and build social contacts [1]. The World Health Organization (WHO) defines participation as “a person's involvement in a life situation” ([2]; p. 15). Children's participation at home, at school, and in the community relates to their overall development, well-being, and quality of life [1, 3]. In addition to family, the outcome of a child's social, psychological, and economic well-being is greatly influenced by school experiences [4]. A child's development involves participation in increasingly complex activities which enable them to acquire the basic skills and competencies necessary for their general development and successful transition into adulthood [1].

Being with peers, being involved in things happening at school, and sense of belonging are requirements of children's participation needs [5]. One key factor for the full participation of children with special needs is the facilitation and flexibility of personal and environmental adaptations, helping students across a range of abilities to do different tasks in shared settings. Active student-teacher cooperation can enhance the student's experience of participation, especially when the individuals are asked for their own ideas for solutions to adapt activities [5].

Enabling a student's participation at school has been an important focus of pediatric occupational therapy services [6]. In countries with inclusive education systems, including all children in regular schools, occupational therapists support children with disabilities to perform activities that enable them to participate in the educational and social aspects of student life at school [7].

The development of school-based occupational therapy services is influenced by political changes, developments in occupational science, and the way in which occupational therapy services are provided. It is also influenced by changes in the educational systems [7, 8]. In the USA, for example, the Education for All Handicapped Children Act (EHA) [9] stipulates that children with a disability are entitled to free education commensurate with their skills and abilities in an inclusive education system. Occupational therapists in the USA have been involved in the process of inclusive education, as a related service, since that law was enacted [7]. Early research in the USA by Niehues et al. [10] and Case-Smith [11] focused on occupational therapy in this new practice setting. The researchers recognized a need to describe clearly their occupational therapy services in more educationally related terms regarding their role and medical science background, in an educational setting.

Developments in special needs education in Europe, including Switzerland, were sparked by the Salamanca conference conducted by the United Nations in 1994, which recognized the need for working towards schools for all. This initiated a framework for action and defined international guidelines to reinforce access to special needs education, accommodating all children regardless of their physical, intellectual, social, emotional, or linguistic impairments or other conditions ([12]; p. 3). Two years later, the European Agency for Special Needs and Inclusive Education (EASN) acted as the platform for collaboration between different countries to develop the provision of special needs education and to promote the full participation of children with special needs in mainstream education [13]. In Switzerland, a federal law was enacted in 2002, requiring all 26 cantons (federal states of the Swiss confederation) to provide education for and enhance the integration of children with disabilities into mainstream schools as much as possible [14].

An agreement was reached in special needs education to collaborate and standardize structures and procedures throughout Switzerland. Between 2008 and 2011, each canton developed concepts of special needs education [15]. These concepts were designed to integrate children with special education needs into mainstream schools. Where these concepts were not possible, the children were placed either in special classes or in schools that were specialized for certain diagnostic groups. Integrative solutions were preferred over segregation (EDK, 2007). These specialized schools included health-related professionals such as physiotherapists and occupational therapists among their staffs and continue to do so [16]. As in other European countries, educational support disciplines in Switzerland remain divided into two categories: education-based and health-based professionals. Swiss occupational therapists and physiotherapists are still financed and supervised by the health system. Legislation has not yet been passed to either legally or structurally embed physiotherapists and occupational therapists as health professionals in the mainstream education system.

Increasing numbers of children with different disabilities are being integrated into regular classes in Switzerland. In order to find the best solutions to enable students with special needs to participate in school, a number of Swiss occupational therapists have started working in individual settings in the public school system [17].

Different authors from a range of countries have described the shift from a biomedical to an educational model as a key factor for successful occupational therapy school service integration and also highlight the challenges of developing services in schools [6, 8, 10, 11]. Other research reports the challenges of adapting practice to the new context and describing services from a more educational basis [8].

School-based occupational therapy practice is strongly related to educational structures and cultural context [1]. The need for school-based research that is situated within the political, structural, and cultural context of a country has been stressed in different studies [1, 8, 18]. In Switzerland, cultural, political, and social factors differ in many ways from those of other countries where most current school-based occupational therapy research has been conducted. Integrating children with special needs into regular schools is relatively new in Switzerland compared with other countries, where extensive research has been undertaken. With the recent changes in Swiss legislation that are aimed at integrating children with special needs into mainstream schools, a gap in knowledge concerning the application of school-based practice has been identified.

With a focus on enabling the participation of students with special needs in the changing school system in Switzerland, occupational therapists are entering an emerging practice field. They must reason and reflect on their practice to explore and develop innovative approaches in this evolving environment [19]. A first step towards acquiring knowledge based on empirical research involves gaining access to practitioners' experiences and their reflections on this emerging practice [20]. This study provides a rare insight into the work of Swiss occupational therapists in the emerging area of school-based practice to discover how they enhance the participation of children with special needs in mainstream schools in Switzerland.

## 2. Method

This study was aimed at exploring Swiss pediatric occupational therapists' practice experiences and clinical reasoning when working with children with special needs in the school context.

The ontological and epistemological positions of this study are, respectively, relativism and social constructivism, where the social world is continuously constructed and reconstructed as human beings interact with each other [21]. Narrative theory implies that the person to whom a story is told and its physical context form part of the constructed findings [22, 23]. Through thematic analysis of eventful narrative data, researchers discover categories, draw relationships, and describe common themes that appear across the narrative data of all participants, thereby reducing the stories to their common elements, and by doing so they create knowledge [24, 25].

### 2.1. Study Design

The design of the study employed a narrative approach which, according to Josephsson and Alsaker [26], gives insight into participants' thought processes and experiences. This is particularly relevant to the participants' developing practice in a new field of mainstream schools in Switzerland. Data that are presented in the form of accounts provide information about connected events and have a temporal sequence and an intentional quality [25]. This approach to analysis through coding facilitates discovering categories, drawing relationships, and describing themes that appear across the accounts of all participants, discovering common elements and thus creating new knowledge [24].

### 2.2. Participants

Due to the political change in mainstream schools, including children with special needs, in Switzerland having occurred relatively recently, there were only a small number of therapists working in this field. The study sample was gathered using purposive sampling with predefined inclusion and exclusion criteria [27].

The Swiss Association of Occupational Therapists (EVS) holds no official register of practitioners who work in mainstream schools (EVS, 2015). Therefore, in order to recruit participants, the researchers identified potential volunteers by various methods: by accessing occupational therapist practitioners' quality circles of the Swiss association (EVS), by approaching presenters at the Swiss congress for occupational therapists, through pediatric continuing education channels, and through the first author's personal network. Possible participants were contacted by email, which described the research project and outlined the inclusion and exclusion criteria. The inclusion criteria were that the practitioners must have worked in mainstream kindergartens or primary schools for a minimum of 6 months and be fluent in German or English. Therapists working in any other function apart from occupational therapy were excluded.

Five therapists meeting the criteria were identified and contacted by phone or Skype. This exchange gave the participants and the researcher the opportunity to become acquainted with one another and to pose additional questions concerning the projects and the practical aspects of further procedures. Occupational science and occupational therapy are committed to diversity and culture [28]. Therefore, five participants from different geographical and language regions of Switzerland were selected, including one participant from the Italian-speaking canton, adding one English interview to the data.  Table 1 presents an overview of their demographic and work-related data.

The time and place of the interviews were scheduled to the participants' convenience. Two weeks prior to the interview, detailed written information was sent by post, including an invitation to contact the researcher if there were any further questions.

### 2.3. Ethical Considerations

According to Schweizerische Ethikkommission für die Forschung am Menschen [29] (the Swiss ethics committee), because this research did not involve investigating people's health and bodily function, no ethical approval was required.

Since the number of therapists working in the school setting in Switzerland is relatively small, there is an enhanced potential for identifying participants involved in this research project. For reasons of confidentiality, the names of the participants have been changed and descriptions of their workplaces have been omitted.

### 2.4. Data Collection

The outline of the interviews was structured according to Küsters [30], starting with an initiation talk, followed by the main narration, the first and second investigative talks, and concluding with interview reflections. A pilot interview with a colleague who met the inclusion and exclusion criteria was conducted to test the interview guide and procedure. Feedback from the interviewed colleague and reflections were discussed, with small changes made to the interview process and the role of the researcher.

Prior to the interview, the participants were invited to ask any further questions concerning the study, and informed consent was obtained at this point. Four of the participants chose to be interviewed in their private practices, whereas the fifth interview took place in a private meeting room of an educational establishment. The interviews were audiotaped and lasted between 55 and 75 minutes. Field notes were composed immediately after the interviews.

### 2.5. Analysis

The analysis procedure used an inductive approach and was achieved according to the six phases outlined by Braun and Clarke [31]. During phase one, the first author transcribed the interviews and familiarized herself with the data by reading through the data set and by taking notes. Four participants spoke in Swiss German dialects, while one interview was conducted in English. Swiss German is a spoken language, and since there are several dialects, a standardized form does not exist. The dialects were, therefore, transcribed into Standard German, a process familiar to Swiss people.

To ensure accuracy in wording and punctuation, the recordings were listened to again and compared to the transcripts. In order to receive a homogeneous database, the English interview was transcribed in English and then translated into German by the first author. A bilingual Anglicist verified the translation and accuracy of content according to cross-language considerations described by Cigdem et al. [32].

In phase two, the data were coded according to their identifying features [31]. Identified codes were related and compared to features across the data set. In phase three, the codes were grouped in subthemes and related to each other in the search for overarching themes. Working with different trails of interpretation, subthemes and related codes were reviewed and restructured, resulting in the identification of three themes described as phase four [26, 31, 33]. In phase five, themes and subthemes were defined and named, and in the final phase, the themes and subthemes were narrated to a report [31].

At the time of coding of the final transcript, no new themes were emerging. As this study was designed as an exploration of an emerging practice field, caution has to be paid to claim saturation [34].

### 2.6. Rigor and Validity

The credibility and dependability of the study design and findings were promoted by ensuring accuracy in transcription through additional relistening and in-depth reading, to become very familiar with the data. A journal was kept for field notes and for reflecting on the researcher's role during the data gathering and analysis processes [35]. To further promote rigor, the interview guide and procedure were reviewed by peers and piloted, and analysis was carried out by using a well-described analysis procedure and by journaling of the audit trail [35, 36].

The trustworthiness of the findings was enhanced by discussion and reflection with peers from the interpretative analysis group at different stages of the analysis, by discussion with peers more experienced in research, and by applying member checking [35].

## 3. Findings

**Figure 1:**
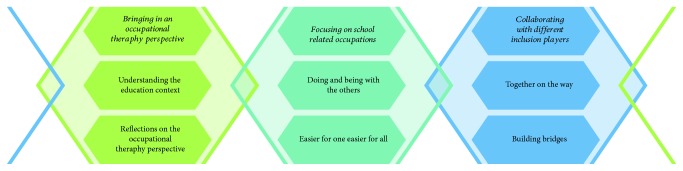
Visual illustration of the themes and related subthemes.

Three main themes were identified in the data: “bringing in an occupational therapy perspective” illustrates the participant's adaptation to and reflections on the changing nature of the education context. “Focusing on school-related occupations” describes the core focus of their work with the children on the one hand and specific school occupations on the other. The third theme, “collaborating with different inclusion players,” derives from the specific cultural and political situation. Each theme comprises two subthemes that are highlighted by quotations from the interviews. Even though each theme and subtheme has specific traits, they interrelate in a dynamic way as they are retrieved from multiple and diverse aspects of the participant's emerging practice, as shown in Figure 1.

### 3.1. Bringing in an Occupational Therapy Perspective

For all the participants, the specific occupational therapy perspective they brought to the field in relation to the education context and how it contributed to the children's participation was fundamental. This was mentioned in terms of “understanding the education context” and “reflections on their occupational therapy perspective.”

### 3.2. Understanding the Education Context

All the participants expressed a need to get to know and understand the children and the teachers' education context in order to bring in a perspective of occupational therapy and thereby complement the education context. Their motivation to learn about life in classrooms and at school is illustrated in their reported commitment to be actively involved in integrating children with special needs into mainstream schools.

The participants saw the school setting in a broad context, with many possibilities for most effectively supporting their clients. To make the most of these possibilities, the participants had to learn and understand everyday life occupations and daily routines in the local schools and individual classroom situations.

More specifically, getting to know the teachers and other people from the children's social environment and seeing the physical environment were two of the key factors in understanding the context. With the recent move in Switzerland towards integrating children with special needs, the participants acknowledged a need to learn about challenging situations for children, teachers, and support teams. They attributed challenges to their lack of experience in working with education professionals and their lack of experience in implementing special education concepts into their practices. In Sue's words,

“I am an occupational therapist and I am going where life is happening, and if it doesn't happen in specialized schools anymore, then I'll follow the child! … The school team lack experience with children with physical disabilities.”

To gain an even deeper understanding, the participants stressed the importance of listening carefully to teachers and learning to communicate in their language.

Lisa said,

“We have to speak the same language as the other members of the team.”

The participant's incentive to understand and learn more about the education context in transition is determined by their clients' needs, reflecting the diagnostic groups they see most, but also appears to be linked to personal interest and work experience.

### 3.3. Reflections on the Occupational Therapy Perspective

Participants perceived their occupational therapy perspective as complementary to the integrative school setting. The participants' main reflections of their specific occupational therapy perspective were as follows: looking at situations in a client-centered way in cooperating with the child's or parents' view, acknowledging children's resources and capabilities, and understanding environmental aspects and the processes and motor skills required to perform school-related tasks. The participants appeared to draw on their medical background knowledge to facilitate understanding on how specific diagnostic traits may have influenced a child's occupational performance and behavior.

A thorough understanding of the child's abilities and special needs appeared to lay the foundation for their therapeutic reasoning. To achieve that understanding, four of the five participants saw the children they worked with in a one-to-one and classroom setting. They described aiming to facilitate classroom staff understanding of a student's specific abilities and limitations.

“…to direct the focus differently, not only on the results, but also on the effort required by the child to produce the results as they are now.” (Cathy)

Hearing the children's and their parents' voices and aiming to bring their view to the teachers' attention, Mary stated,

“I do that often - I listen to what the children say and tell it to the teachers, for example, about that girl who wanted to re-join the class circle and was maybe just too shy to tell the teacher.”

### 3.4. Focusing on School-Related Occupations

The participants understood occupation as their core business and adopted different occupation-centered approaches. In their practical experience, a child's occupation and his or her participation are closely related, in the sense of “doing and being with others.” A more specific view of school-related occupation describes the theme as “easier for one easier for all.”

### 3.5. Doing and Being with Others

The participants used occupations which corresponded to children's capabilities in order to influence their occupational performance and their position in the classroom or at school. For example, a child with a physical impairment liked to play table tennis, so the bat was adapted. His skills developed to a level that he became “…the table tennis king during morning break.” This enabled the boy to do the things he wanted to do and be with others. Sue commented,

“Yes, we have to find something that the child is really good at and can really participate in. I think you find something with every child.”

The dynamics of interaction and participation in activities outside the classroom changed in a positive way when the activities were linked to a child's interest, and capabilities were made possible by adapting tools or creating an environment conducive to participation.

To work on identified occupational performance problems, the participants used a small group or separate setting outside the classroom. Such a setting seemed to allow the participants to create time and space to develop children's skills and listen to the children's views and needs. Depending on age and setting, the children were facilitated in the transfer of their current skills in the classroom and reflected on these. Mary reported a comment made by a student who attended a small group setting and applied the skills in a larger setting with others, for example, in the classroom and at school in general:

“He said himself, he learns much more about the doing … What does he really have to do and how does he want to do it? … Also about social competencies, which he can apply at school.”

George supported one boy with autism spectrum disorder in developing the self-organization skills needed to engage in constructional games. George explained that other children became interested in joining their small “construction group” playing construction games and they then began playing with this boy. This in turn altered his position within the class system, doing and being together with other children from his class, engaged in the same occupation.

These examples seemed to illustrate, in diverse ways, how the “doing” influenced the “being with others” and therefore the children's participation.

### 3.6. Easier for One Easier for All

Four of the five participants addressed issues of specific students in their class following a referral by a pediatrician. The participants stated that they focused on the children's occupational performance and involved their teachers in developing strategies related to the classroom. They observed that the teachers then transferred these strategies to other children with similar problems.

Handwriting as occupation is one example in which the participants accessed a broader school context. While George developed preschool handwriting training for one specific child, he introduced it to the whole class.

Cathy reported examining ways to simplify the diverse handwriting approaches in Switzerland. She reported that since working in a school for children with special needs, she argued for an easier approach for students with special needs. She stated that she is now involved in imparting her knowledge of a simplified handwriting approach to four hundred teachers in mandatory continuing teacher education. The continuous exchange with the teachers' experiences helped her to further her knowledge. This project sparked off another project at the teachers' training college.

“In order for all children to benefit, one could change many things at school to make it easier for everyone and, of course handwriting is one of them.” (Cathy)

Occupations themselves which are related to everyday life at school were described as a possible means of a participant's involvement at school.

### 3.7. Collaborating with Different Inclusion Players

Each participant started their work with a child before that child's integration into regular classes began. From there, they proceeded to “go on the way with different inclusion players” and started to “build bridges” to connect different people and contexts. To develop collaboration and to build bridges appear to take time and play a major role in integrating children successfully.

### 3.8. Together on the Way

The referral practices of the participants and the nature of their involvement at school depended on the local situations, diagnostic groups, and the participant's own resources. Sue, for example, worked as a specialized consultant mainly with children with physical impairments. Her referred clients came from mainstream schools to the specialized school for children with physical impairments, where she worked as a staff occupational therapist. The school acted as a center of expertise for children of this diagnostic group. From this setting, Sue's specialized knowledge and experience were transferred into the mainstream school setting.

In the other settings, the participants entered as private practitioners, aiming to support the teachers and students in their classrooms by developing strategies to enable children's participation in everyday life at school.

Although each participant's setting was different, they all stressed the importance of developing a trusting collaborative relationship with the classroom staff, which required time. Teachers' experiences and preferences are incorporated into the development of these collaborative service strategies. In Sue's words,

“The most important thing, we have to go on the way with these people … each setting is different, and as a therapist you can only observe, see what is there and work from there.”

Interventions were described as most effective when active complementary collaboration could be developed. Flexibility in their approaches has been identified as one key factor. Another key factor is identifying things which do not work.

### 3.9. Building Bridges

Since Swiss therapists are independent of the school system, they perceived themselves to be in a neutral position, allowing them to build bridges. Participants appeared to bridge the gaps between home and school and students and teachers and to serve as catalysts to connect a larger support environment outside the school to the children's individual school setting.

George's key questions were as follows:

“Who needs support, what kind of support can be considered, who renders the support, how is it implemented and who finances it?”

Being directly on the school site on a weekly basis appeared to make Cathy's services more available to all teachers and children, which she reported as enhancing collaboration and opening informal paths of communication, bridging education and therapy knowledge. Cathy and Mary considered this to be more beneficial to the children and their classroom teachers.

Participants also reported that seeing parents and children outside the school setting provided the time and space to listen to their needs and successes. By communicating back to the schools, participants seemed to bridge the gap between the different inclusion players from the home and school environments. The participating therapists viewed linking occupations and participation, in collaboration with the education staff, as an element that bridges the health and education systems. Bridges span from personal, structural, and system levels.

## 4. Discussion

Through the participants' experiences described above, this study provides insight into the practice of occupational therapists in mainstream schools in Switzerland. It contributes to the knowledge base of the strengths and limitations of this emerging area of practice.

The three main themes illustrate different aspects of this work. The first theme, “bringing in an occupational therapy perspective,” provides insight into the education context and how practitioners complement this context by contributing their specific perspective. The “focusing on school-related occupations” theme centers around different occupation-based approaches and their effect on one or more children. The third theme, “collaborating with different inclusion players,” identifies different aspects of the practitioners' collaboration with different people involved in educating, supporting, and parenting the children.

It is argued that the themes interrelate in a dynamic way in order to bring the participants' occupational therapy services and perspectives to enhance cross-professional understanding in an educational environment. Participants focused on school-related occupations that were meaningful to the children and teachers, facilitating participation through occupation. They collaborated with different inclusion players and built bridges from school and home and between education and health systems to implement intervention.

School-based occupational therapists in the USA, Australia, New Zealand, and Canada are part of education teams with the common goal to fully include children in student life at school [1, 7, 8, 18, 37], whereas the study findings indicate that in Switzerland the current practice proposes to integrate the children “as much as possible” (Eidgenoessische Konferenz der Erziehungsdirektoren, EDK, 2007). This potentially indicates differences in the structure and intention for the inclusion of Swiss children with disabilities and the position of school-based Swiss occupational therapists compared to those of other countries.

Service delivery models such as Partnering for Change [37] or Response to Intervention [38] have been developed in inclusive school environments with occupational therapists as part of the education team. Both these models are based on the collaboration of educators, parents, students, and therapists. The study findings suggest that, because school-based occupational therapists in Switzerland are relatively new, these therapists have worked in fields outside education and consequently bring to the field a network of agencies outside the educational system, which could enhance the services delivered to the children. In the subtheme “together on the way,” participants illustrated how they adapted their “set of key players” with respect to the requirements of the specific situation and built bridges for collaborative working and experience and knowledge exchange. The exchange of knowledge is part of service delivery models.

At this early stage of integrative education in Switzerland, schools and the school curriculum still tend to be planned and organized for children without disabilities and those who are physically independent and have age-appropriate cognitive and social skills, and most classroom teachers have little or no experience in working with children with special needs [15]. The study findings indicate the participants' acknowledgment that their skills, abilities, and networks could support not only the child with special needs but also educators or caregivers, supporting the call of Mundhenke et al. [39] for the diversity of support required for effective service delivery.

The theme “bringing in an occupational perspective” presents the participants' reflections on their specific contribution towards working with children and collaborating with teachers in a client-centered way. Participants described listening to the children's views and needs outside the classroom setting and discovering a child's motivation and strengths as important approaches to help therapists, parents, and teachers to see the children from a broad occupational perspective. This finding supports those of Bonnard and Anaby [40], Hasselbusch and Penman [41], Missiuna et al. [37], Sayers [42], and Villeneuve [43]. Although this approach allowed the therapists to create time and space to work on individual needs, the sociopolitical and cultural context of the values of both classroom and individual settings may be lost, and there may be a need for further research to explore the specific needs of the individual child and the needs of the extended social or physical environment [44].

Children with special needs state that school staff have a limited understanding of how disability can affect their schoolwork and impact on their participation [39]. At this early stage of integrative education in Switzerland, schools and the school curriculum still tend to be planned and organized for children without disabilities and those who are physically independent and have age-appropriate cognitive and social skills. Most of the classroom teachers had very little or no experience in working with children with special needs [15].

The process of altering views and offering alternative interpretations is described as reframing, one of the therapists' most important tools in the education context [10, 11, 41, 45]. By including children's and parents' views as part of their specific perspective, the participants took reframing a step further. Students report that their experience of participation was facilitated when they were included in finding solutions for adapting activities to their abilities [5]. Reframing not only appeared to alter expectations regarding the outcome of children's performance but also seemed to direct intervention strategies in adapting the environment or tasks throughout the occupational therapy process [11, 37, 41]. The findings that participants valued children's insights and suggestions in discovering solutions and giving them the opportunity to express preferences [46] have been linked to a more favorable educational outcome [47]. Little appears known about the processes required to enable children from a young age through to adulthood and their families to express preferences in finding solutions for participation at school, which the authors argue is an area worthy of future research.

Recent views on participation move away from an individualistic perspective of participation, where the focus lies on the individual apart from the environment, towards seeing the individual and their participation context as one ongoing cycle of change [48]. Recognizing the importance of the social environment in developing “collaboration with different inclusion players” appeared an essential component in the participants' evolving school-based practice. This finding supports previous research which highlights the importance of developing working relationships based on trust and equality and engaging all key players in problem-solving processes; both of which require time and seem vital for successful school-based occupational therapy services [11, 37, 41].

In their clinical work, the participants took the teachers' experiences and preferences into consideration. During this process, the practitioners reported being supportive and respectful of school staff requests and suggestions for solutions [37, 41, 42]. The apparent flexibility in their approaches and in identifying things that do not work appeared as key factors the participants used to negotiate solutions in a collaborative way [37, 41].

All the participants originally practiced and had contacts outside the mainstream school system, providing them with a network of multiprofessional colleagues and services outside the education context. They appeared to draw on these experiences, build bridges, and work with colleagues from the schools to encourage the exchange of experiences and knowledge. Knowledge translation can be seen as the act of exchanging information, knowledge, and resources with colleagues in order to learn from each other [37]. Regarding going “together on the way,” the participants acknowledged the changing nature of the school context. At the same time, they acknowledged the importance of a continuous exchange between the different key players, while adapting an interdisciplinary approach to their services to benefit the students' participation in school.

With the “focus on occupation,” the participants applied different approaches to address participation issues. In the sense of the student's “doing and being with others,” Sue and George tapped into the children's interests, resources, and capabilities to influence their position in the class or at school. Sue adapted the child's table tennis bat and George created a construction group, with both using different means to adapt to the environment. This could be seen as the capability approach which is based on resources and aims to equalize children's capability sets by eliminating restrictions, such as adapting to the environment and at the same time including children's choices [49]. Rodger [3] advocates the shift from disability to ability and adopts a strength-based practice approach as a stepping stone towards children's full participation in school, allowing them to do and be with others. However, being outside the education system, occupational therapists need to find alternative avenues to contribute to strength-based practice and, in Anna's words, find “something in every child that they are good at.”

### 4.1. Strengths and Limitations

Although the strength of this study is its contribution to knowledge concerning the current occupational therapists' practice in Swiss mainstream schools, it must also be recognized that this is a changing environment and the findings of this study may quickly become obsolete. The process of integrating children with special needs into mainstream education is still in the early stages, and therefore, the participants are experiencing changing times in special needs education strategy implementation.

The study is limited by the method of one interview per participant. This offers a good scoping of experiences but does not provide the opportunity to delve into the phenomena. Research conducted over a longer period of time, follow-up interviews, and additional field observations might have led to findings that provided a deeper insight into the participant's experiences [26].

Although the study was successful in exploring the experiences of Swiss occupational therapists who work in mainstream education arenas, it could be argued that it is limited by its homogeneity. If the study had included the views of other professions involved, a broader picture could have been appreciated and the role of the occupational therapist as a new player explored.

Finally, the strength of the study rests with its recruitment process. Switzerland is a multilingual country with Swiss German dialects that do not exist in an official written form. In order to not only overcome language barriers but also include dialects of the Swiss language diversity and therefore regions, this study purposefully recruited participants across this diversity to include cross-lingual aspects and experience in its findings.

## 5. Conclusion

The findings of this study provide insight and knowledge concerning the development of Swiss occupational therapy practice in mainstream schools, taking political and cultural aspects of the special needs education context in Switzerland into consideration.

In the Swiss context, as in many other European countries, health and education services are cooperating to enable children's participation in mainstream schools. The study findings of three themes and six subthemes interrelate in a dynamic way. They indicate how occupational therapists as part of the health system are exchanging knowledge and working with the education system in a meaningful way.

The findings highlight three aspects of the participants' accounts of their work in an emerging practice field. By “bringing in an occupational therapy perspective,” practitioners adapted their specific perspective to improve the facilitated understanding of children's occupational performance and behavior which, in turn, altered expectations and directed intervention strategies. A “focus on occupation” highlighted that practitioners centered their intervention on different school-related occupations by addressing environmental, personal, or occupational aspects, changing single or multiple variables to facilitate children's participation. The third theme illustrated how participants collaborated with different inclusion players, transferred knowledge, and built bridges to connect different contexts. Collaboration based on an egalitarian and trusting relationship with different inclusion players is one key factor for successful school-based practice. Research has established that children's involvement in finding solutions to resolve issues facilitates their participation experience. To enable children to fully participate in all aspects of being a student in mainstream schools in Switzerland and other European countries, research is needed connecting the different perspectives of inclusion players within the specific school environment.

## Figures and Tables

**Table 1:** Description of participants.

Name	Age	Work experience	% work in schools	Core diagnostic groups	Work settings other than schools
Mary	52 yrs.	12 yrs.	15-25%	ADHD	PP
Cathy	47 yrs.	23 yrs.	40%	ASD, DCD, ADHD	PP, SRP
George	45 yrs.	18 yrs.	15-20%	ASD	PP, SES
Lisa	42 yrs.	18 yrs.	15%	ADHD, DCD, LD	PP, SRP
Sue	52 yrs.	28 yrs.	20%	PD	SES

Note: ADHD = attention-deficit hyperactivity disorder; DCD = developmental coordination disorder; ASD = autism spectrum disorder; LD = learning disability; PD = physical disability; PP = private practitioner; SRP = school-related projects; SES = special education setting.

## Data Availability

The data has been collected by the first author in the written form of the transcribed interviews. The data is available on request, by contacting the first author Angelika Echsel (angelika.echsel@bluewin.ch).

## References

[1] Rodger S., Ziviani J. (2006). *Occupational Therapy with Children: Understanding Children’s Occupations and Enabling Participation*.

[2] World Health Organisation (2001). *International Classification of Functioning, Disability and Health (ICF)*.

[3] Rodger S. (2010). *Occupation-Centred Practice with Children: A Practical Guide for Occupational Therapists. Chichester, United Kingdom*.

[4] Law M., Petrenchik T., Ziviani J., King G., Rodger S., Ziviani J. (2006). Participation of children in school and community. *Occupational Therapy with Children*.

[5] Asbjørnslett M., Hemmingsson H. (2008). Participation at school as experienced by teenagers with physical disabilities. *Scandinavian Journal of Occupational Therapy*.

[6] Clark G. F., Clark G. F., Chandler B. E. (2013). Best practice in school occupational therapy interventions to support participation. *Best Practice for Occupational Therapy in Schools*.

[7] Clark G. F., Chandler B. E. (2013). *Best Practices for Occupational Therapy in Schools*.

[8] Simmons Carlsson C., Hocking C., Wright-St Clair V. (2007). The “why” of who we are: exploring the “culture of practice” of Ministry of Education, special education occupational therapists and physiotherapists. *Kairaranga*.

[9] *Education for All Handicapped Children Act of 1975*.

[10] Niehues A. N., Bundy A. C., Mattingly C. F., Lawlor M. C. (2016). Making a difference: occupational therapy in the public schools. *The Occupational Therapy Journal of Research*.

[11] Case-Smith J. (1998). *Occupational Therapy: Making a Difference in School System Practice*.

[12] United Nations Educational, Scientific and Cultural Organisation (UNESCO) (1994). *The Salamanca Statement and Framework for Action on Special Needs Education; World Conference on Special Needs Education*.

[13] European Agency for Development in Special Needs Education (2011). *Key Principles for Promoting Quality in Inclusive Education. Recommendations for Practice*.

[14] Eidgenössische Konferenz der Erziehungsdirektoren (2007). Interkantonale Vereinbarung über die Zusammenarbeit im Bereich der Sonderpädagogik. *Heiden*.

[15] Hollenweger J. (2011). Development of an ICF-based eligibility procedure for education in Switzerland. *BMC Public Health*.

[16] Ergotherapeutinnen Verband Schweiz (2017). *EVS Ergotherapeutinnen Verband Schweiz*.

[17] Gewerkschaft Pflichtschullehrerinnen und Pflichschullehrer Österreich, GÖD-APS, Dachverband Lehrerinnen und Lehrer Schweiz, LCH, & Verband Bildung und Erziehung Deutschland, VBE (2014). *Berliner Erklärung zur Inklusion*.

[18] Swinth Y., Spencer K. C., Jackson L. L. (2007). *Occupational Therapy: Effective School-Based Practices within a Policy Context*.

[19] Holmes W. M., Scaffa M. E. (2009). The nature of emerging practice in occupational therapy: a pilot study. *Occupational Therapy in Health Care*.

[20] Carrier A., Levasseur M., Bédard D., Desrosiers J. (2010). Community occupational therapists’ clinical reasoning: identifying tacit knowledge. *Australian Occupational Therapy Journal*.

[21] Finlay L., Ballinger C. (2006). *Research for Allied Health Professionals-Challenging Choices*.

[22] Josephsson S., Asaba E., Jonsson H., Alsaker S. (2006). Creativity and order in communication: implications from philosophy to narrative research concerning human occupation. *Scandinavian Journal of Occupational Therapy*.

[23] Molineux M., Richard W. (2003). Storied approaches to understanding occupation. *Journal of Occupational Science*.

[24] Bailey D. M., Jackson J. M. (2003). Qualitative data analysis: challenges and dilemmas related to theory and method. *The American Journal of Occupational Therapy: Official Publication of the American Occupational Therapy Association*.

[25] Polkinghorne D. E. (1995). Narrative configuration in qualitative analysis. *International Journal of Qualitative Studies in Education*.

[26] Josephsson S., Alsaker S., Nayar S., Stanley M. (2015). Narrative methodology: a tool to access unfolding and situated meaning in occupation. *Qualitative Research Methodologies for Occupational Science and Therapy*.

[27] DePoy E., Gitlin L. N. (2011). *Introduction to Research Understanding and Applying Multiple Strategies*.

[28] World Federation of Occupational Therapists (2010). Diversity matters: guiding principles on diversity and culture. https://www.wfot.org/resources/diversity-and-culture.

[29] Schweizerische Ethikkommission für die Forschung am Menschen (2015). https://www.swissethics.ch/.

[30] Küsters I. (2009). *Narrative Interviews*.

[31] Braun V., Clarke V. (2006). Using thematic analysis in psychology. *Qualitative Research in Psychology*.

[32] Cigdem E., Fathi M., Squire C., Flick U. (2014). Narrative analysis. *The Sage Handbook of Qualitative Data Analysis*.

[33] Kohler Riessman C. (2008). *Narrative Methods for Human Science*.

[34] Faulkner S. L., Trotter S. P., Matthes J., Davis C. S., Potter R. F. (2017). Theoretical saturation. *The International Encyclopedia of Communication Research Methods*.

[35] Collingridge D. S., Gantt E. E. (2008). The quality of qualitative research. *American Journal of Medical Quality*.

[36] Miles M. B., Huberman M. A., Johnny S. (2014). *Qualitative Data Analysis: A Methods Sourcebook*.

[37] Missiuna C. A., Pollock N. A., Levac D. E. (2012). Partnering for change: an innovative school-based occupational therapy service delivery model for children with developmental coordination disorder. *Canadian Journal of Occupational Therapy*.

[38] Ardoin S. P., Witt J. C., Connell J. E., Koenig J. L. (2005). Application of a three-tiered response to intervention model for instructional planning, decision making, and the identification of children in need of services. *Journal of Psychoeducational Assessment*.

[39] Mundhenke L., Hermansson L., Sjöqvist Nätterlund B. (2010). Experiences of Swedish children with disabilities: activities and social support in daily life. *Scandinavian Journal of Occupational Therapy*.

[40] Bonnard M., Anaby D. (2016). Enabling participation of students through school-based occupational therapy services: towards a broader scope of practice. *British Journal of Occupational Therapy*.

[41] Hasselbusch A., Penman M. (2008). Working together: an occupational therapy perspective on collaborative consultation. *Kairaranga*.

[42] Sayers B. R. (2008). Collaboration in school settings: a critical appraisal of the topic. *Journal of Occupational Therapy, Schools, & Early Intervention*.

[43] Villeneuve M. (2009). A critical examination of school-based occupational therapy collaborative consultation. *Canadian Journal of Occupational Therapy*.

[44] Hollenweger J. (2018). Creating learning opportunities together, instead of teaching here and supporting there: outlining a procedure for joint planning in inclusive settings. *Schweizerische Zeitschrift Für Heilpädagogik*.

[45] Bundy A. C. (2002). Using sensory integration theory in schools: sensory integration and consultation. *Sensory Integration: Theory and Practice*.

[46] Hemmingsson H. (2010). Making children’s voices visible: the school setting interview (SSI). *Kairaranga*.

[47] Wehmeyer M. L., Schalock R. L. (2001). Self-determination and quality of life: implications for special education services and supports. *Focus on Exceptional Children*.

[48] Jones M., Hocking C., McPherson K. (2017). Communities with participation-enabling skills: a study of children with traumatic brain injury and their shared occupations. *Journal of Occupational Science*.

[49] Morris C. (2009). Measuring participation in childhood disability: how does the capability approach improve our understanding?. *Developmental Medicine & Child Neurology*.

